# Emirati Women’s Knowledge about the Menopause and Menopausal Hormone Therapy

**DOI:** 10.3390/ijerph17134875

**Published:** 2020-07-06

**Authors:** Linda Smail, Ghufran A. Jassim, Khawla I. Sharaf

**Affiliations:** 1 Department of Mathematics and Statistics, Zayed University, Dubai 19282, UAE; 201316162@zu.ac.ae; 2 Royal College of Surgeons in Ireland-Medical University of Bahrain, Busaiteen 15503, Bahrain; gjassim@rcsi-mub.com

**Keywords:** menopause, Emirati women, knowledge, healthcare policy, menopausal hormone therapy (MHT)

## Abstract

The aim of this study was to investigate the knowledge of Emirati women aged 30–64 about menopause, menopausal hormone therapy (MHT), and their associated health risks, and additionally, to determine the relationships between Emirati women’s knowledge about menopause and their sociodemographic and reproductive characteristics. A community-based cross-sectional study was conducted of 497 Emirati women visiting five primary healthcare centers in Dubai. Data were collected using a questionnaire composed of sociodemographic and reproductive characteristics, menopause knowledge scale (MKS), and menopause symptoms knowledge and MHT practice. The mean menopause symptoms knowledge percentage was 41%, with a standard deviation of 21%. There were significant differences in the mean knowledge percentage among categories of education level (*p* < 0.001) and employment (*p* = 0.003). No significant differences in the knowledge percentages were found among categories of menopausal status. “Pregnancy cannot occur after menopause” was the statement with the highest knowledge percentage (83.3%), while the lowest knowledge percentages were “risk of cardiovascular diseases increases with menopause’’ (23.1%), “MHT increases risk of breast cancer’’ (22.1%), and “MHT decreases risk of colon cancer’’ (13.9%). The knowledge of Emirati women about menopause, MHT, and related heart diseases was very low; therefore, an education campaign about menopause and MHT risks is needed to improve their knowledge for better coping with the symptoms.

## 1. Introduction

Menopause is a natural biological process leading to a transition from a reproductive to a non-reproductive state experienced universally by all women due to ovarian failure. The age at which menopause occurs is generally between 45 and 55 years, with an average onset of 51 years [1].

Based on the 2010 United Arab Emirates (UAE) census [2], the estimated life expectancy for Emirati women living in Dubai is 39.4 years for those currently aged 45–49 years, and 34.4 years for those aged 50–54 years. Based on these life expectancies, women are expected to live at least one-third of their lives beyond menopause, which will have tremendous implications on the healthcare system and its policies in the future [3].

There is a wide range of menopause research exploring age at menopause, attitudes and knowledge of menopause, and menopausal hormone therapy (MHT) [4,5,6,7,8]. Menopause research has been a game changer for women’s lives. Most studies in the Arab world reported a younger age at menopause, low to average knowledge about menopause, and very low knowledge and usage of MHT [9,10,11,12,13]. To the best of our knowledge, only three studies have been published that surveyed women’s knowledge of menopause and MHT-related health risks in different emirates of the UAE [14,15,16].

Ibrahim et al. [14] reported that 48% of women were using MHT for menopausal symptoms, 45% of women thought that MHT was a good option, and 25 % had negative attitudes towards MHT. Eman et al. [15] studied the knowledge and attitudes towards menopause and MHT in Abu Dhabi and found that the knowledge about MHT was very limited, in fact only 38% had heard of MHT before. Saima et al. [16] reported that the women’s knowledge about menopause was very poor and varied significantly with level of education and nationality.

To our knowledge, there were no available studies that investigated knowledge about menopause and MHT among Emirati women exclusively. All published studies in this topic included women from different nationalities residing in the UAE. The proposed study will be the first to investigate knowledge about menopause and MHT among Emirati women. It will have a great impact on decision makers regarding healthcare policies of women’s health.

The objectives of this study were as follows: first, to investigate the knowledge of Emirati women in Dubai about menopause, MHT, and their associated health risks, and second, to examine the relationship between Emirati women’s knowledge about menopause and their socio-demographic information and reproductive characteristics.

## 2. Materials and Methods

### 2.1. Study Design and Sample

A community-based cross-sectional study was conducted among a random sample of 497 Emirati women aged 30 to 64 years from the Emirate of Dubai. The study participants were recruited from primary healthcare centers in the Emirate of Dubai. The sample size was determined based on a power of 85% and a significant level of 5%.

### 2.2. Sampling Method

The public healthcare system in Dubai is run by the Dubai Heath Authority (DHA). It has a total of twenty primary healthcare centers (PHC) in the Emirate of Dubai [17]. Sampling was done first by selecting five PHC out of the twenty-five centers, and then selecting a simple random sample of women from each PHC. Medically induced menopausal women, pregnant, and breast-feeding women were excluded because of amenorrhea or menstrual irregularities which will result in difficulties in grouping them into different menopausal categories. In addition, women with surgical menopause (hysterectomy) and those using progesterone and intrauterine contraceptive device (IUCD) were excluded as well.

### 2.3. Study Instruments

Women were interviewed directly by a trained nurse using a questionnaire that had two parts. The first part contained the socio-demographic variables and reproductive characteristics. Reproductive characteristics consisted of an assessment of menopausal status, which is defined as the following: women who had regular menstrual periods in the last 3 months were classified as Premenopausal, women who indicate their periods had become irregular but they had a period in the last 12 months were classified as Perimenopausal, and women who indicate they had not had a period in the last 12 months or longer were classified as Postmenopausal. Data about oral contraceptive pills’ use (current, past, and never) and perceived general health (very good, good, not so good, and bad) were collected.

The second part consisted of the menopause symptoms knowledge questions, and Menopause Knowledge Scale (MKS). The MKS was developed by Appling and colleagues [18] and validated by psychometric analyses using factor analysis, item analysis, and internal consistency reliability estimates. Cronbach’s alpha (α) equaled 0.81. In the original MKS, there are eight items regarding knowledge of menopause and six items regarding MHT knowledge and their health risks. Based on different literature [9,10,11,12,13,14,15,16], MHT use and knowledge are reported at low levels. Further, women in the Middle East region prefer to use natural remedies rather than medications to manage symptoms. Therefore, we added one item about knowledge of menopause to the existing 8 items to make a total of 9 items. Another 9 items regarding MHT knowledge were added to the existing six items to make a total of 15 items. As a result, the MKS has 24 items in total (9 items on menopause knowledge and 15 items on MHT knowledge). MKS summed scores range from 0 to 24 on the MKS, with lower scores indicative of more knowledge about menopause (agree = 1, disagree = 2). Reliability analysis was performed on the modified MKS questionnaires with a Cronbach’s alpha of 0.858.

### 2.4. Ethical Consideration

We sought ethical approval from the Zayed University research and ethics committee (REC number: ZU17_004_F) and the Dubai Health Authority medical research committee (REC number: DSREC-02/2017-15). Additionally, permissions from the selected PHC centers were obtained. The selected Emirati women were invited to participate in the study on a voluntary basis. Participants were assured of confidentiality and anonymity.

### 2.5. Statistical Analysis

The collected data were coded, entered, and analyzed using the Statistical package SPSS, version 25 (IBM Corp., Armonk, NY, USA). Descriptive statistics were computed for all variables. The percentage of correct answers from each respondent to all items was calculated and called the ‘knowledge percentage’. The newly created variable knowledge percentage served as the dependent (outcome) variable in the study and was used for subsequent data analyses. Socio-demographic and reproductive variables served as independent variables. One-way analysis of variance (ANOVA) or the independent sample t-test were carried out to test the equality of population means across the categories of each independent variable (predictor) depending on the independent variable number of categories. Pearson’s linear correlation coefficient was computed to assess the linear relationship between each of the outcome variables and each of the quantitative independent variables. Multiple linear regression analysis was used to predict each of the outcome variables using the following predictors: educational level, marital status, employment, age, menopausal status, and use of oral contraceptive pills. Statistical tests with *p*-values < 0.05 were considered statistically significant.

## 3. Results

In this study, 497 women consented to participate. The mean and standard deviation of the respondent’s age was 42.06 ± 8.7 years, with a median age of 40 years and a range of 34. The Emirati women’s knowledge about the mean age at menopause was 49.9 ± 4.6, with a median age of 50 years old and a range of 35.

Table 1 presents the characteristics of the women in the study sample. The majority (74.3%) were married and premenopausal (70.8%). A total of 41.1% were university undergraduate students. The majority of participants (93.4%) had ‘never used hormone replacement therapy’.

Table 2 illustrates the knowledge of the participants regarding 13 menopause symptoms. The symptoms that were recognized by more than half of the participants were ‘hot flashes’ (55.1%), ‘depression’ (67.4%), ‘mood swings’ (72.6%), ‘insomnia’ (56.9%), ‘feeling more tired than usual’ (55.1%), ‘weight gain’ (51.1%), and ‘irritability’ (61%). Forty percent knew that ‘vaginal dryness’ is one of the menopausal symptoms.

Table 3 represents the answers for the participants on their knowledge of menopause and MHT and their associated health risks. As the results demonstrate, the ‘risk of cardiovascular diseases increases with menopause’ was correctly answered by 23.1%. More than half (59.4%) agreed that ‘menopause is accompanied by hot flushes’. Moreover, approximately 83.3% of women knew that ‘pregnancy cannot occur after menopause’. 

Twenty-two percent of the participants agreed that ‘MHT can increase risk of breast cancer’, and 27.6% were aware that ‘MHT can decrease the risk of osteoporosis’, the statement of ‘MHT can increase the risk of heart disease’ was agreed by 18.5%, and 28.2% knew that ‘MHT can reduce vasomotor symptoms’. Over 29% believe that ‘MHT has many complications and side effects’ and 24% agree that the ‘risks of taking MHT outweigh the benefits.’ In addition, more than 61% of the participants believe that ‘natural approaches are better than MHT.’ 

Table 4 shows that there were significant differences in the mean knowledge percentages among the different categories of education level (*p* < 0.0001) and employment (*p* = 0.003). Table 4 shows that illiterate women had the lowest knowledge percentage and university graduates had the highest. Also, employed women had a higher knowledge percentage than unemployed women. 

Table 5 displays the results of the multiple linear regression model for knowledge percentage. It contains the predictors of education, marital status, employment, age, use of oral contraceptive pills (OCP), and menopausal status, whereas the knowledge percentage is considered the dependent variable. Over 10% of the variation in the knowledge percentage (R^2^ = 0.103) was explained by the variables included in the multiple regression model, which proposes that there are other variables than those included in the study that may explain the variations in the knowledge percentage. The only two predictors that had a significant effect on the knowledge percentage in the model were education level (*p* < 0.001) and age (*p* = 0.006).

In the simple linear regression, where smoking was the only predictor, it explained only 1% of the variation in attitudes, while when menopausal status was the only predictor, it explained only 1.6%. Additionally, education level explained approximately 1.4% of the variation in knowledge.

## 4. Discussion

### 4.1. Knowledge of Menopause, MHT, and Their Associated Health Risks

In our study, the mean knowledge percentage of menopause and MHT was 40.56, with a standard deviation of 20.68, which is an indication of a low level of knowledge about menopause and MHT, especially about associated heart diseases. In fact, the lowest knowledge percentages were about the statements: “ Risk of cardiovascular diseases increases with menopause’’ (23.1%), “MHT increases risk of breast cancer’’ (22.1%), “MHT can increase the risk of heart disease’’ (18.5%), and “MHT decreases the risk of colon cancer’’ (13.9%).

These results are similar to what was found in the study of Jassim et al. [13] but with even lower knowledge percentages than Bahraini women. In fact, 33.3% of Bahraini women correctly answered the statement “MHT can increase the risk of cardiovascular diseases’’ and 40% for “Risk of cardiovascular diseases increases with menopause’’ whereas it was 18% and 23.1% respectively, for Emirati women.

It is important to note that 95.6% of the Emirati women participants had never used MHT and therefore were not counseled about the benefits and risks of MHT, which explains the low knowledge percentage about MHT and related heart diseases. Another factor is that the majority of the sample were premenopausal women, so they had not been through menopause yet.

This is similar to what was found in the Egypt study of Loutfy et al. [10], where 91% of the women never heard of MHT. Additionally, the results reported in References [15,16] are similar to our study—the knowledge of menopausal symptoms was low, with only 38% having heard of MHT in Reference [15] and 21% in Reference [16].

On the other hand, the highest knowledge percentage of correct answers was about the statement “pregnancy cannot occur after menopause” (83.3%), the same statement was the highest answered by Bahraini women in the study of Jassim et al. [13] (75.8%). This similar result is an indication that women in both countries agree that the only thing they are sure of about menopause is they will not be reproductive anymore because fertility is a priority to women and men in both countries, and more generally in this region of the world. It is important to note that the highest percentage can be explained because women in this region have a negative cultural view about menopause because it is considered as the end of a woman’s life as they are not reproductive anymore, which is evident by the Arabic name given to menopause, which is “desperate age”.

These results are also comparable with what was found in the study of Lam et al. [19], where it was found that the majority of postmenopausal Hong Kong Chinese women have a low level of knowledge about MHT. In our study, less than a third of the Emirati women were aware that MHT can reduce vasomotor symptoms (28.2%), which is similar to results announced in the study of Lam et al. [19] about Hong Kong Chinese women (23.5%).

### 4.2. Factors Affecting Menopause and MHT Knowledge

In our study, the knowledge percentage of Emirati women about menopause and MHT was significantly associated with education level and employment status. This is consistent with the findings of Bahraini women in the study of Jassim et al. [13] and with a study by Appling et al. [18] that found a direct link between women’s knowledge about menopause and their education and employment status. Women who had a higher education degree or employment status had more knowledge about menopause than less educated or unemployed women, which was also reported by Saima et al. [16].

A study of Lam et al. about Hong Kong Chinese women [19] found that women with more symptoms, who had used MHT, and those with higher education levels and higher family income, had better knowledge about MHT. Additionally, in the same study, the symptom scores of premenopausal women were significantly lower than those of perimenopausal women but were comparable with those of postmenopausal women.

However, in our study, there was no significant relationship between menopause knowledge and menopausal status, marital status, and the use of oral contraceptive pills, which is similar to what was found among Bahraini women in the study of Jassim et al. [13].

Also, 10.3% of the variation in the knowledge percentage was explained by the variables included in the multiple regression model, which proposes that there are variables other than those included in the study that may explain the variations in the knowledge percentage. The only two predictors that had a significant effect on knowledge were education level (*p* < 0.001) and age (*p* = 0.006).

These results may suggest that menopause knowledge is more related to personal characteristics than menopause itself and that more factors may influence Emirati women’s attitudes towards menopause that should be investigated in future studies.

### 4.3. Premenopausal versus Peri- and Post-Menopausal Women

In this study, seven knowledge statements were significantly related to menopausal status: ‘Menopause is accompanied by hot flushes’ (*p* = 0.002), ‘Risk of osteoporosis increases with menopause’ (*p* = 0.010), ‘Menopause can have harmful consequences if not treated’ (*p* = 0.005), ‘MHT can reduce vasomotor symptoms’ (*p* = 0.015), ‘MHT can decrease the risk of osteoporosis’ (*p* = 0.023), ‘MHT is a good solution, if you have symptoms’ (*p* = 0.004), and ‘MHT is good for preventing age-related health problems’ (*p* = 0.020).

In our study, postmenopausal women knew more than premenopausal women about hot flashes and risk of osteoporosis, but more premenopausal women knew that MHT can reduce vasomotor symptoms and that MHT is a good solution if there are symptoms. This may be explained by the fact that postmenopausal women have already experienced the symptoms and are more aware of their occurrence than premenopausal women who did not go through the symptoms yet. On the other hand, perimenopausal women knew more than postmenopausal women about the fact that MHT is good for age-related health problems. Additionally, postmenopausal women knew more than premenopausal women about the fact that menopause can have harmful consequences if not treated. This may be explained by the fact that post- and peri-menopausal women answered based on their own experiences, but premenopausal women had not yet experienced menopause.

## 5. Strengths and Limitations

This is the first study exploring Emirati women’s knowledge about menopause and the first one taking place in the Emirate of Dubai. This study will open the door for more research on menopause and will be a basis for healthcare decision makers to develop policies to improve Emirati women’s knowledge about menopause, MHT, and their associated health risks.

As the sample was drawn only from healthcare centers in Dubai, the results cannot be generalized to all Emirati women populations.

In this study, we did not consider variables such as number of children, age at menarche, and income, and it may be worth looking at their effects on menopause and MHT knowledge. Additionally, we did not include questions related to menopause and religion. This may also have an impact on the Emiratis women’s knowledge about menopause and MHT.

## 6. Conclusions

Emirati women’s knowledge about menopause and MHT is relatively low. Educational level and employment status were the only two variables significantly associated with menopause and MHT knowledge. Therefore, there is a need for raising the awareness regarding menopause symptoms and management options.

## Figures and Tables

**Table 1:** Characteristics of the study sample (N = 497). SD = standard deviation.

Variable	Level	N	%
**Educational level**	Illiterate	16	3.3
	Primary School	31	6.4
	Preparatory School	43	8.8
	Secondary School	178	36.6
	University Undergraduate	201	41.4
	University Graduate	17	3.5
**Employment**	Yes	220	44.6
	No	273	55.4
**Marital status**	Single	79	16.1
	Married	364	74.3
	Divorced	32	6.5
	Widowed	15	3.1
**Menopausal status**	Premenopausal	352	70.8
	Perimenopausal	65	13.1
	Postmenopausal	80	16.1
**Smoking**	Yes	17	3.6
	No	450	96.4
**Use of oral contraceptive pills (OCP)**	Current users	15	3.1
	Past users	161	32.9
	Never used	313	64
**Use of menopausal hormone replacement therapy (MHT) (N = 145)**	Current users	13	9.0
	Past users	20	13.8
	Never used	112	77.2
**Self-rated health**	Bad	2	0.4
	Not so good	48	9.7
	Good	284	57.1
	Very Good	163	32.8
**Age**		Mean = 42.06	SD = 8.7

**Table 2:** Knowledge of Menopause Symptoms (N = 497).

Symptoms	Yes	No	Do Not Know
Hot flashes	55.1	12.7	32.2
Night sweats	47.9	15.7	36.4
Depression	67.4	10.3	22.3
Mood swings	72.6	6.8	20.5
Insomnia	56.9	13.3	29.8
Feeling more tired than usual	55.1	13.3	31.6
Weight gain	51.1	15.7	33.2
Having difficulty concentrating	40.2	20.9	38.8
Breast pain	40.8	16.5	42.7
Irritability	61	9.3	29.8
Vaginal dryness	40.2	13.5	46.3
Leak of urine when coughing, sneezing, or laughing	35.8	18.7	45.5
Hair thinning	43.9	13.9	42.3

**Table 3:** Percentage of participants who agreed to individual items on the menopause and menopausal hormone therapy (MHT) knowledge scale (N = 497).

Statement	Agreement Percentage
Menopause is due to a decrease of female hormones	67%
Menopause occurs when menstruation stops	79.3%
Pregnancy cannot occur after menopause	83.3%
Menopause occurs when ovaries stop functioning	63%
Menopause is accompanied by hot flushes	59.4%
Risk of cardiovascular diseases increases with menopause	23.1%
Risk of osteoporosis increases with menopause	54.1%
Risk of depression increases during the menopause period	60.2%
Menopause can have harmful consequences if not treated	40.6%
MHT replaces hormones decreasing during menopause	34%
MHT can reduce vasomotor symptoms	28.2%
MHT can increase the risk of heart disease	18.5%
MHT can decrease the risk of osteoporosis	27.6%
MHT increases risk of breast cancer	22.1%
MHT decreases risk of colon cancer	13.9%
MHT is a good solution, if you have symptoms	35.8%
MHT is appropriate for some women	60.6%
MHT is to be avoided	29.8%
MHT is in some cases good for preventing age-related health problems	27.2%
MHT has many complications and side effects	29.6%
Natural approaches are better than MHT	52.5%
Risks of taking MHT outweigh the benefits	24.7%
MHT prevents obesity	14.1%
MHT improves hot flashes	24.9%

**Table 4:** Knowledge percentage by independent variables (N = 497).

Variable	Level	Mean	SD	*p*-Value ^a^
Educational level	Illiterate	55.9	10.9	<0.0001
	Primary School	54.5	12.1	
	Preparatory School	53.6	9.8	
	Secondary School	50.7	11.2	
	University Undergraduate	47.5	12.3	
	University Graduate	45.4	11.9	
Menopausal status	Premenopausal	50.3	11.9	0.286
	Perimenopausal	47.8	11.8	
	Postmenopausal	49.6	11.5	
Marital status	Single	48.5	12.6	0.379
	Married	50.4	11.8	
	Divorced	48.1	11.1	
	Widowed	47.3	12.1	
Use of oral contraceptive pills (OCP)	Current users	47.3	11.9	0.614
	Past users	49.5	10.9	
	Never used	50.1	12.3	
Employment	Yes	41.2	12.7	0.003
	No	51.2	10.9	

^a^*p*-Value based on analysis of variance (ANOVA) or independent *t*-test. Lower score indicates higher knowledge.

**Table 5:** Final general linear model with parameter estimates for knowledge percentage.

Variable	Level	β	SE	95% Confidence Interval	*p*-Value
Corrected model	-	-	-		<0.001
Intercept	-	53.388	6.873	(39.9, 66.9)	<0.001
Educational level	Illiterate	13.330	4.342	(4.8, 21.9)	<0.001
	Primary School	10.958	3.872	(3.3, 18.6)	
	Preparatory School	10.958	3.610	(2.0, 16.2)	
	Secondary School	5.826	3.153	(−0.4, 12.0)	
	University Undergraduate	2.293	3.091	(−3.8, 8.4)	
	University Graduate	0	0		
Marital status	Single	0.362	3.683	(−6.9, 7.6)	0.624
	Married	2.110	3.347	(−4.5, 8.7)	
	Divorced	0.750	3.923	(−7.0, 8.5)	
	Widowed	0	0		
Employment	Yes	1.612	1.272	(−0.9, 4.1)	0.206
	No	0	0		
Age	-	−0.246	0.088	(−0.4, −0.1)	0.006
Use of oral contraceptive pills (OCP)	Current users	−6.003	3.301	(−12.5, 0.5)	0.098
	Past users	−1.639	1.183	(−4.0, 0.7)	
	Never used	0	0		
Menopausal status	Premenopausal	0.480	2.067	(−3.6, 4.5)	0.291
	Perimenopausal	−2.084	2.247	(−6.5, 2.3)	
	Postmenopausal	0	0		

R^2^ = 0.103. SE: Coefficients Standard Error
